# Optimization of the Cohesion Index in the SeDeM Diagram Expert System and application of SeDeM Diagram: An improved methodology to determine the Cohesion Index

**DOI:** 10.1371/journal.pone.0203846

**Published:** 2018-09-13

**Authors:** Isaac Nofrerias, Anna Nardi, Marc Suñé-Pou, Joost Boeckmans, Josep Maria Suñé-Negre, Encarna García-Montoya, Pilar Pérez-Lozano, Josep Ramón Ticó-Grau, Montserrat Miñarro-Carmona

**Affiliations:** 1 Pharmacy and Pharmaceutical Technology, and Physical Chemistry Department, Faculty of Pharmacy and Food Sciences, University of Barcelona, Barcelona, Spain; 2 Department of Molecular Biology, Institute of Parasitology and Biomedicine “López Neyra” (IPBLN-CSIC), PTS, Granada, Spain; 3 Pharmacotherapy, Pharmacogenetics and Pharmaceutical Technology Research Group, IDIBELL-UB, Barcelona, Spain; University of Sheffield, UNITED KINGDOM

## Abstract

In this study, we suggest optimizing the methodology to determine the Cohesion Index (Icd) in order to avoid mistaken characterizations due to powder bulk density. For this purpose, five different excipients, with different bulk densities and of different chemical nature, were compressed at different heights. Their compression and their tablet characterization enable establishing a powder weight for compression in accordance with its bulk density. Therefore, the resulting tablet will have a height within a defined range of heights where it has no critical effects on its hardness. Then, the impact of this optimization is shown in a formula development, one of the main SeDeM’s applications. A mathematical equation was used to calculate the theoretical amount of excipient to formulate the API according to both methodologies. The compression results demonstrate that the characterization with the NM-Icd is more accurate than the previous one while preserving its simplicity.

## Introduction

The pharmaceutical industry aims to manufacture medicines at the optimum cost. To achieve this goal, the methodology selected must lead to robust and optimized products in terms of output. It is known that direct compression is one of the most desirable ways to produce tablets because the lead times for manufacturing are low, and requirements for equipment, solvents, and residues are reduced. However, the process simplicity requires that the powdery blend meets with the adequate parameters for direct compression technology. The blend has to display adequate densities, powder flow, compressibility and the final tablet must be stable over the time. Moreover, the active pharmaceutical ingredient should conform more than 1% of the final formulation. Otherwise, it can yield to a segregation during the compression. Therefore, it is great of interest in a pharmaceutical development to be able to identify and select which formulas gather these requirements, and the rejection of those that need previous processes, such as granulation [1–3]. The SeDeM methodology was designed aiming this goal.

**Fig 1:**
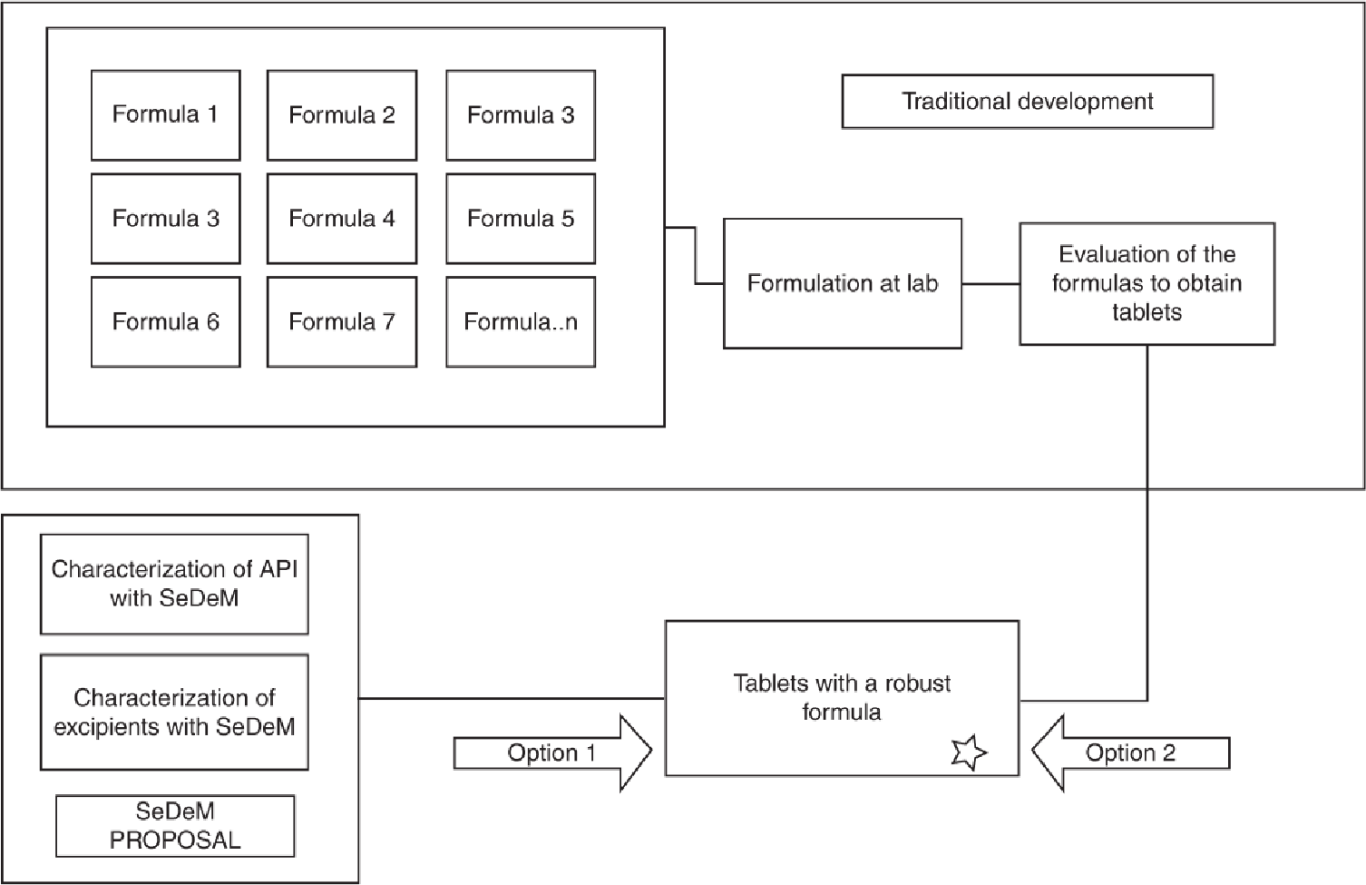
Comparison of strategies for development.

The SeDeM Methodology [4] is a pharmaceutical method applicable in tablet formulation studies. It provides information about the suitability of powdery substances for direct compression, such as active ingredients or excipients. This information indicates the degree to which substances can be successfully compressed by means of direct compression technology. The SeDeM Methodology enables detecting the weakest properties of the powder, which must be corrected, to facilitate the formulation of the final blend for direct compression. As a result, it saves time and accelerates the development of the formula (see Fig 1).

Furthermore, the SeDeM Methodology is also a useful tool for studying the reproducibility of the process used to manufacture a powder and, consequently, for its validation [5]. As established in earlier studies [4–13] the SeDeM Methodology quantitatively and experimentally characterizes the powder’s parameters, which allows obtaining the necessary information about a substance’s suitability for obtaining tablets using direct compression technology. The parameters considered are listed below:

- Bulk Density (Db)
- Tapped Density (Dt)
- Inter-particle Porosity (Ie)
- Carr Index (IC)
- Cohesion Index (Icd)
- Hausner Ratio (IH)
- Angle of Repose (α)
- Powder Flow (t”)
- Loss on Drying (%HR)
- Hygroscopicity (%H)
- Particle Size (%Pf)
- Homogeneity Index (IӨ)

These parameters are determined by means of the SeDeM Diagram method, based on known equations [5], and duly validated with reproducible experimental tests, as shown in Table 1.

**Table 1:** Parameters and equations used in SeDeM methodology.

Incidence	Parameter	Symbol	Unit	Equation
Dimension	Bulk Density	Da	g/mL	Da = M/Va
	Tapped Density	Dc	g/mL	Dc = M/Vc
Compressibility	Inter-particle Porosity	Ie	-	Ie = (Dc-Da)/(Dc*Da)
	Carr Index	IC	%	IC = (Dc-Da)/Dc*100
	Cohesion Index^a^	Icd	N	Experimental
Flowability/Powder flow	Hausner Index ratio	IH	-	IH = Dc/Da
	Angle of Repose	(α)	°	Tgα = h/r
	Powder Flow	t”	s	Experimental
Lubricity/Stability	Loss on Drying	%HR	%	Experimental
	Hygroscopicity	%H	%	Experimental
Lubricity/Dosage	Particles <50 μm	%Pf	%	Experimental
	Homogeneity Index^b^	(Iϴ)	-	Iϴ = Fm/100+ΔFmn^a^

^a^ Hardness (N) of the tablets obtained with the tested product, alone or blended with lubricants if highly abrasive.

^b^ Determines particle size in accordance with the percentages of the different particle size fractions

Once the parameters of the SeDeM Diagram were established, the acceptable numerical limit values for each of the 12 study parameters were determined. These values are shown in Table 2.

**Table 2:** Limit values accepted for the SeDeM Diagram parameters and conversion factor to convert each parameter into radius values (r).

Incidence	Parameter	Limit value (V)	Radius (r)	Factor applied to v
Dimension	Bulk Density	0–1 g/mL	0–10	10v
	Tapped Density	0–1 g/mL	0–10	10v
Compressibility	Inter-particle Porosity	0–1.2	0–10	10v/1.2
	Carr Index	0–50%	0–10	v/5
	Cohesion Index	0–200 N	0–10	v/20
Flowability/Powder flow	Hausner Index ratio	3–1	0–10	(30-10v)/2
	Angle of Repose	50–0 (°)	0–10	10-(v/5)
	Powder Flow	20–0 (s)	0–10	10-(v/2)
Lubricity/Stability	Loss on Drying	10–0 (%)	0–10	10-v
	Hygroscopicity	20–0 (%)	0–10	10-(v/2)
Lubricity/Dosage	Particles <50 μm	50–0 (%)	0–10	10-(v/5)
	Homogenity index	0–2 x 10^−2^	0–10	(5x10^2^)v

If all radius values are 10, the SeDeM diagram creates a twelve side regular polygon as a result from attaching the different radii by linear segments. The previous experimental values are converted in radius values and the figure created describes the product characteristics and each parameter indicates if the powder is suitable for direct compression. The SeDeM diagram used in this study is formed by 12 parameters or, in other words, it is formed by 12 sides. In order to determine numerically whether the product is suitable for direct compression or not, the following indexes are calculated:

$$Parameter\;Index\;(IP)=\frac{No.Pt\geq 5}{No.Pt}$$

No. Pt ≥ 5 indicates the number of parameters whose value is equal to or higher than 5, No. Pt: Indicates the total number of parameters studied. The acceptability limit would correspond to: IP≥0.5.

Parameterprofileindex(IPP)=meanrofallparameters

Mean r = mean value of the parameters calculates.

The acceptability limit would correspond to: IPP ≥5

Good compression Index (IGC) is calculated as follows:

GoodcompressionIndex=IPP×f

Where f is the reliability factor and is calculated as follows:

$$f=\frac{Polygon\;area}{Circle\;area}$$

The acceptability limit would correspond to: IGC ≥ 5

If the IGC or a high number of parameters are below 5, the SeDeM diagram indicates that the powdery substance is not suitable for direct compression technology and several troubles may be involved during its compression.

In addition, as a methodology for characterizing substances in relation to their viability in direct compression, the SeDeM Diagram Expert System may be considered as being an open system in terms of the number of parameters applied and the optimization of these parameters. In the stablished SeDeM system (see Table 1), the sole parameter which measures powder cohesion is the Cohesion Index (Icd) [4,9]. According to the current methodology to determine Icd, it is required to make 5 oval convex tablets of 1 gram with a 19x10 mm punch format with an eccentric press [4,13]. The Cohesion Index radius value (r) is calculated from the average of these 5 tablet crushing strength measurements (resistance to crushing), which it is also known as hardness in the pharmaceutical literature [14]. The force required to cause the tablet failure is the most common measurement due to its simplicity and rapid application as a pharmaceutical control. This parameter is fairly relevant since it indicates if the powder is able to make proper bonds when a compression force is applied. Therefore, a good Cohesion Index radius value is linked with an adequate compactability [15]. As the final tablet was established as a 1 (±0.05) gram, the factors involved in the final tablet dimensions depend on the following factors:

- Minimum punch distance: In the compression of a perfect plastic material, the tablet’s height would be the minimum distance between punches. Since compression is done at maximum compression force, the distance between punches is the smallest allowed for each powder. Then, this distance depends on the powder’s compressibility, such as its particle rearrangement ability, inter-particle porosity, morphology or deformation behaviour (brittle, plastic, elastic) [16–18].
- Elastic recovery: During compression, the powder stores part of the whole energy as elastic forces. After compression, the tablet undergoes elastic recovery, which implies tablet expansion. Then, the final tablet dimensions depend on the chemical nature of the powder [16,19,20].
- Bulk density: The powder’s bulk density has a critical impact on tablet dimensions. For the same weight, powders with high bulk density occupy less volume in the die, which means thinner tablets, and vice-versa.

Regarding these factors, the Cohesion Index may not be accurate for powders with a low or a high bulk density. On the one hand, high density powders cannot be compressed at 1 gram due to the risk of breaking punches, or they result in thin tablets that break easily. This is the case of dicalcium phosphates. Although they are described in the literature as DC filler/binders [16,21], they show low radius values for the Icd [12].On the other, it has been observed that it was not possible to reach 1 gram for some substances whose bulk density was quite low. There was not sufficient room in the die. In these cases, it is not viable to make a 1 gram tablet and get a feasible radius value.

The aim of this study is to improve the methodology which determines the Cohesion Index parameter in order to prevent deviations due to the powder’s bulk density, i.e., to obtain more accurate and verified experimental results. For this purpose, five excipients of different bulk densities (DC fillers: Emcompress^®^, Lactosa Fast Flo^®^, Comprecel^®^ 302, Kollidon^®^ Va 64 and Microcel^®^ 102) are used to develop the new methodology to determine the Cohesion Index. These excipients have different chemical nature, different compression behaviour and different density [12,21,22]. The different density allows studying how the bulk density of the powder affects the tablet hardness whereas the different chemical nature and compression behavior facilitate extrapolating the results to other excipients. The compression of the excipients at different tablet heights and their comparison with the tablets obtained to make the SeDeM diagram provides information about the influence of the excipient’s bulk density in the Icd determination. With these results, the improved methodology to determine the Icd is proposed (NM-Icd). The improved weight adjustment according to the bulk density increases the reliability of the Icd radius value, where high bulk density excipients require making heavier tablets and low bulk density excipients require making lighter tablets. Finally, the impact of this modulation is evaluated preparing and compressing one mixture of MCC/API/Lubricant. The API selected is one which usually displays a bad Cohesion Index. Therefore, in this study is used as a tracer API. The selected excipient to correct the API’s lack of cohesion is a MCC because MCCs tends to display a low bulk density but it is described as one of the most useful fillers to DC due to its excellent compactability [16]. The amount of each formula’s component was calculated according to the mathematical equation in the design of direct compression tablet formulation [6] and the formula is compressed by DC. The characterization of the tablets is used to evaluate if the NM-Icd is more accurate than the previous methodology to determine the Icd.

## Materials and methods

### Powders

The material under study consists of 5 excipients for direct compression, three excipients which comprise the SeDeM lubricant mix, and one API. All of them are listed below: Colloidal silicon dioxide Batch 16H22-T05 (FAGRON IBÉRICA, Terrasa (Barcelona), Spain), Comprecel^®^ 302 Batch C1403108-S (Ming Thai Chemical Co., LTD. Taiwan, R.O.C.), Emcompress^®^ Batch 9003 (JRS Pharma GGmbH & Co. KG, Rosenberg, Germany), Kollidon^®^ Va 64 Batch 51799616150 (Basf, Ludwigshafen, Germany), Lactose Fast Flo Batch 8513060861(Seppic, Paris, France), Magnesium Estearate Batch 14F25-B02-301892 (FAGRON IBÉRICA, Terrasa, Spain), Microcel^®^ 102 Batch 125001008 (Blanver, Farmoquímica, Sao Paulo, Brasil), Sulfadimethoxine Batch 753098–0, Talc Ph. Eur. Batch 14L04-T01 (Fagron Ibérica, Terrasa, Spain).

### Powder characterization

The procedure for the product characterization of these substances involves determining the 12 parameters of the SeDeM Diagram [4–6]. The methods indicated in the pharmacopoeias were applied wherever possible. When the methods were not available, a system based on the common practice used in pharmaceutical research and specifically adapted for the SeDeM Diagram was proposed [4,5,13]. The methods used for each test are described in Protocols IO, dx.doi.org/10.17504/protocols.io.r27d8hn. The methods are also described below:

Bulk density (Da): The method is described in Section 2.9.34 of Eur. Ph. (Ph Eur, 2011) Tapped density (Dc): The method is described in Section 2.9.34 of Eur. Ph. (Ph Eur, 2011) The volume taken is the value obtained after 2500 strokes using a settling apparatus with a graduated cylinder (voluminometer).

Inter-particle porosity (Ie) of the powder mixture (Font, 1962) is calculated from the following equation: Ie = Dc-Da/Dc×Da.

Carr index (IC%) (Córdoba et al, 1996; Rubinstein, 1993; Torres & Camacho, 1991; Wong, 1990). The method is described in Section 2.9.34 of Eur. Ph. (Ph Eur, 2011) This is calculated from Da and Dc as: IC = (Dc-Da/Dc)100

Cohesion index (Icd): This index is determined by compressing the powder, preferably in an eccentric press. 3.5% of the following mixture is added to the mix: talc 2.36%, Aerosil® 200 0.14% and magnesium stearate 1.00%. The mean hardness (N) of the tablets is calculated.

Hausner ratio (IH) (Ph Eur, 2011; Rubinstein, 1993). The method is described in Section 2.9.34 of Eur Ph (Ph Eur, 2011). This is calculated from Da and Dc as: IH = Dc/Da

Angle of repose (α) (Rubinstein, 1993, Muñoz, 1993). The method is described in Section 2.9.36 of Eur Ph (Ph Eur, 2011). This is the angle of the cone formed when the product is passed through a funnel with the following dimensions: height 9.5 cm, upper diameter of spout 7.2 cm, internal diameter at the bottom, narrow end of spout 1.8 cm. The funnel is placed on a support 20 cm above the table surface, centred over a millimetre-grid sheet on which two intersecting lines are drawn, crossing at the centre. The spout is plugged and the funnel is filled with the product until it is flush with the top end of the spout when smoothed with a spatula. Remove the plug and allow the powder to fall onto the millimetre sheet. Measure the four radii of the cone base with a slide calliper and calculate the mean value (r). Measure the cone height (h). Deduce α from tan(α) = h/r.

Flowability (t″): The method is described in Section 2.9.16 of Eur. Ph (Ph Eur, 2011). It is expressed in seconds and tenths of a second per 100 grams of sample, with a mean value of three measurements.

Loss on drying (%HR): This is measured by the method described in 2.2.32 in Eur. Ph (Ph Eur, 2011). The sample is dried in an oven at 105°C ± 2°C, until a constant weight is obtained.

Hygroscopicity (%H): Determination of the percentage increase in sample weight after being kept in a humidifier at a relative humidity of 76% (±2%) and a temperature of 22°C± 2°C for 24 h.

Percentage of particles measuring <50 μm (%Pf): Particle size is determined by means of the sieve test following the General method 2.9.12 of Eur. Ph. (Ph Eur, 2011). The value returned is the % of particles that pass through a 0.05-mm sieve when vibrated for 10 min at speed 10 (CISA vibrator).

Homogeneity index (Iθ): This is calculated according to the General method 2.9.12 of Eur. Ph (Ph Eur, 2011). To determine particle size by means of the sieve test, the grain size of a 100g sample is measured by subjecting a sieve stack to vibration for 10 min at speed 10 (CISA vibrator). The sieve sizes used are 0.355 mm, 0.212 mm, 0.100 mm and 0.05 mm. The percentage of product retained in each sieve is calculated and the amount that passes through the 0.05mm sieve is measured. The percentage of fine particles (<50 μm) (%Pf) was calculated as described above. Note that if this percentage is higher than that calculated in the complete sieve test, it is because some of the particles become adhered to the product retained in the sieves during the grain-size test, and the percentage of <50 μm particles found may be lower than the true figure. The following equation is then applied to the data obtained.

$$I\theta =\frac{Fm}{\begin{array}{c} 100+(dm-\mathrm{dm}-1)Fm-1+(\mathrm{dm}+1-dm)Fm+1+(dm-\mathrm{dm}-2)Fm-2+\\ +(\mathrm{dm}+2-dm)Fm+2...+(dm-\mathrm{dm}‑n)+(dm+\mathrm{dm}+n)Fm+n \end{array}}$$

Where: Iθ, Relative homogeneity index. Particle-size homogeneity in the range of the fractions studied; Fm, percentage of particles in the majority range; Fm−1, percentage of particles in the range immediately below the majority range; Fm+1, percentage of particles in the range immediately above the majority range; n, order number of the fraction studied under a series, with respect to the major fraction; dm, mean diameter of the particles in the major fraction; dm−1, mean diameter of the particles in the fraction of the range immediately below the majority range; dm+1, mean diameter of the particles in the fraction of the range immediately above the majority range.

### Influence of powder density in the determination of the Icd of the SeDeM diagram

Five excipients (Emcompress^®^, Lactosa Fast Flo^®^, Comprecel^®^ 302, Kollidon^®^ Va 64 and Microcel^®^ 102) were compressed with the same press and punches used in the SeDeM methodology, a Bonals^®^ (Cornellà de Ll., Spain) continuous eccentric press, provided with 19x10 mm punches. Nevertheless, instead of the established one gram as in the SeDeM methodology [4], tablets were made to different heights: 5.00 mm, 4.00 mm, 3.00 mm, 2.50 mm and 2.00 mm (± 0.20 mm deviation accepted). Compression was always at the highest possible compression force for each excipient [4]. The tablets were characterized: hardness was measured (Dr. Schleuniger Pharmatron, Model 5Y tablet tester), height was measured (Metaltest), and weight was measured with an analytical balance (Mettler Toledo, AB104).

### Statistical analyses

A statistical study was conducted to analyse the results using STATGRAPHICS centurion (Statgraphics Technologies, Inc. The Plains, Virginia) with the ANOVA test and then with the Fisher’s least significant difference (LSD) test, a multiple range contrast test to describe significant differences between groups. The groups that are significantly different are indicated by an asterisk. With this method, there is a 5% of error to assume that two groups are significantly different when the real difference is 0.

### Application of SeDeM diagram: New methodology applied in formula design

The amount of Microcel^®^ 102 needed to correct sulfadimethoxine weakest points (low “Compressibility” parameters) was calculated according to the mathematical equation [6] for the two characterizations performed (one per methodology). The excipient and the drug substance were sieved through an 800 μm sieve and were mixed for 15 minutes at 20 rpm using a biconic mixer (Glatt Labor Tecnic). Then, the SeDeM lubricants mix was sieved through a 600 μm sieve and was added to the obtained mixture, mixing for a further 5 minutes at 20 r.p.m. The characterization of the mixture was performed following the SeDeM Method as described in 2.2.

The mixture was compressed with the same press and punches used in the SeDeM Method, a Bonals^®^ (Cornellà de Ll., Spain) continuous eccentric press, provided with 19x10 mm punches, but automatically. The parameters were adjusted to obtain tablets of 1 gram at the highest possible compression force. The tablets were characterized: mass uniformity and friability (Dr. Schleuniger Pharmatron, FRV 2000) were determined according to their pharmacopoeia monographs [23]. Hardness was also characterized (Dr. Schleuniger Pharmatron, Model 5Y tablet tester).

## Experimental results and discussion

### Powder characterization

First of all, the parameter values were obtained for all the products following the described methodology. Each parameter was determined three times and the mean value was used for radius calculation. Diagram values were calculated by applying the equations in Table 1. The figures obtained were then converted into radii (r), as shown in Table 2. The corresponding parameters and the mean radius values obtained with the samples studied are shown in Table 3. The results slightly differ from previous work [12] but this is due to the use of different batches [5].

**Table 3:** Parameters, incidence means and parametric index.

		SeDeM characterization of DC excipients																			
		Prameters (r)												Mean incidence					Index		
*N*	Excipient	Da	Dc	Ie	IC	Icd	IH	(a)	t"	%HR	%H	%pf	(IO)	Dimens	Compresib	Flowab/Pflow	Lub/stability	Dosage/lubrif	IP	IPP	IGC
1	Microcel 102	3.29	4.21	5.53	4.37	4.87	8.60	3.33	0.00	3.36	9.20	0.00	1.95	3.75	4.92	3.98	6.28	0.98	0.25	4.06	3.86
	Batch: 125001008																				
2	Comprecel 302	4.41	5.83	4.60	4.87	10.00	8.39	4.84	5.92	4.27	8.56	3.67	2.25	5.12	6.49	6.38	6.42	2.96	0.42	5.63	5.36
	Batch:C1403108_S																				
3	Lactose FastFlo	5.74	7.11	2.80	3.85	10.00	8.81	6.03	8.16	6.85	10.00	8.17	3.10	6.43	5.55	7.66	8.43	5.63	0.75	6.72	6.40
	Batch: 8513060861																				
4	Emcompress	7.20	9.02	2.33	4.04	6.57	8.74	5.92	9.27	5.14	9.85	9.16	4.60	8.11	4.31	7.97	7.50	6.88	0.75	6.82	6.49
	Batch: 9003																				
5	Kollidon® Va 64	3.51	4.39	4.76	4.01	3.88	8.75	6.48	8.00	8.18	2.23	7.62	6.15	3.95	4.22	7.64	5.20	6.89	0.50	5.66	5.39
	Batch: 51799616150																				
6	Sulfadimethoxine	6.00	8.11	3.62	5.20	0.00	8.24	4.82	5.55	9.30	10.00	3.59	5.15	7.06	2.94	6.20	9.65	4.37	0.67	5.80	5.52
	Batch: 753098–0																				

### Influence of powder density in the determination of the Icd of the SeDeM diagram

**Fig 2:**
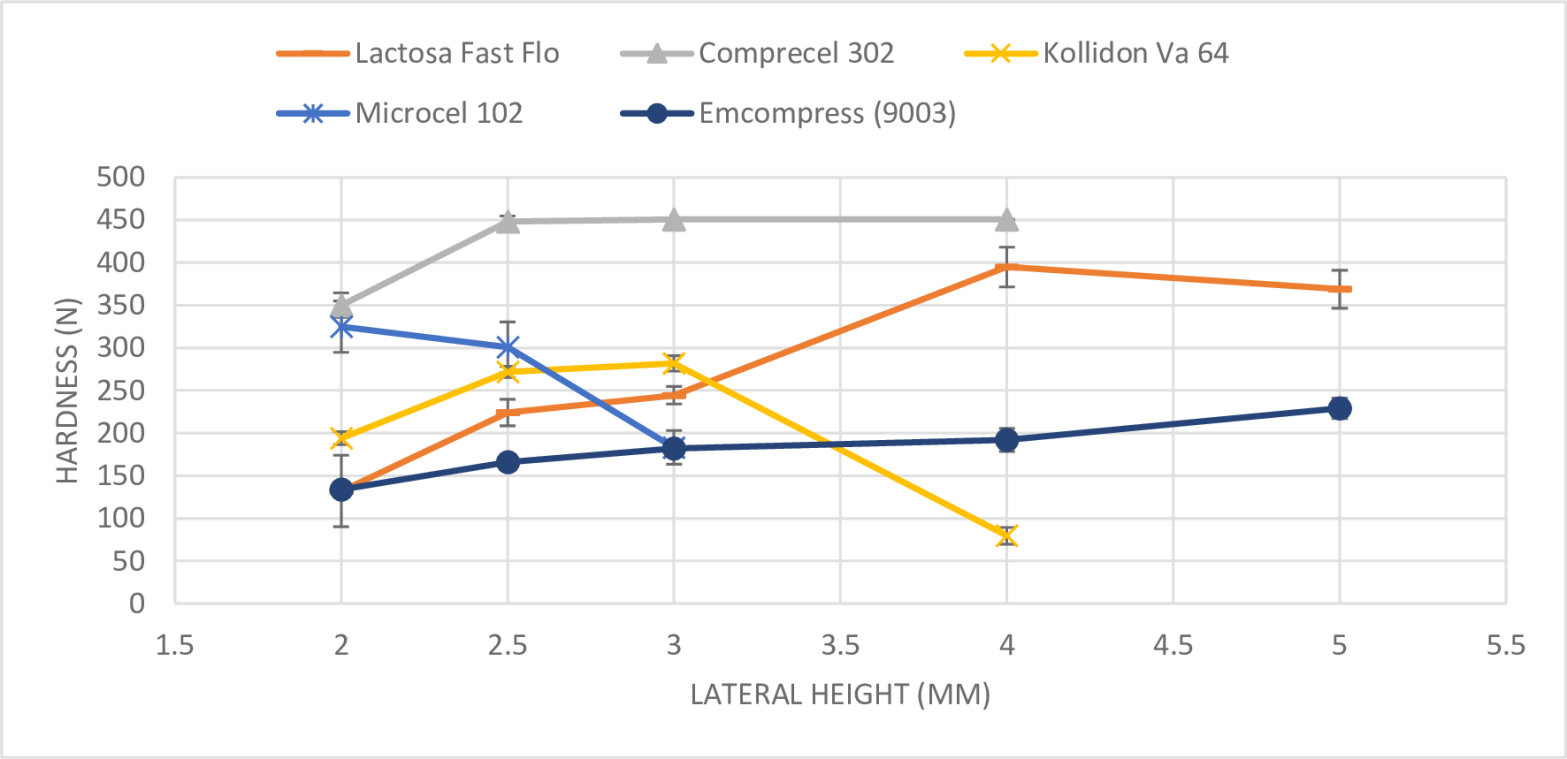
Tablet hardness (N) vs. lateral height of the five excipients compressed.

As it was expected, the five excipients selected display different bulk densities (see Table 3). The powders were compressed fifteen times for each different height in order to know the correct weight to compress concerning the excipient’s bulk density. Then, for each height, tablet characterization was performed following the described methodology so as to ascertain the weight according to the excipient’s bulk density. The hypothesis is as follows: excipients with high bulk density require a greater amount of powder to be compressed than excipients with low bulk density. The mean values for their hardness, weights and heights are shown in Table 4, where they are compared against SeDeM results. In the same way, Fig 2 shows the trend followed for each excipient.

**Table 4:** Mean values from the obtained tablets: Weight, height and hardness.

Height: 2 mm			
Excipient	Weight (g)	Height (mm)	Hardness (N)
Emcompress^®^	1.0556	1.97	134
Lactosa Fast Flo^®^	0.7671	2.17	132
Comprecel^®^ 302	0.7204	2.06	350
Kollidon^®^ Va 64	0.5573	2.06	194
Microcel^®^ 102	0.6944	1.98	325
Height: 2,5 mm			
Excipient	Weight (g)	Height (mm)	Hardness (N)
Emcompress^®^	1.1521	2.41	166
Lactosa Fast Flo^®^	0.8438	2.48	224
Comprecel^®^ 302	0.8244	2.49	448
Kollidon^®^ Va 64	0.6683	2.54	272
Microcel^®^ 102	0.7270	2.46	301
Height: 3 mm			
Excipient	Weight (g)	Height (mm)	Hardness (N)
Emcompress^®^	1.2944	2.91	182
Lactosa Fast Flo^®^	0.9110	3.00	244
Comprecel^®^ 302	0.9449	3.09	≥ 451
Kollidon^®^ Va 64	0.7329	2.87	282
Microcel^®^ 102	0.7537	3.07	183
Height: 4 mm			
Excipient	Weight (g)	Height (mm)	Hardness (N)
Emcompress^®^	1.6584	4.02	192
Lactosa Fast Flo^®^	1.2042	3.96	395
Comprecel^®^ *302*	1.0674	4.07	> 451
Kollidon^®^ Va 64	0.7607	3.96	80
Microcel^®^ 102	N.A.	N.A.	N.A.
Height: 5 mm			
Excipient	Weight (g)	Height (mm)	Hardness (N)
Emcompress^®^	1.9546	5.04	229
Lactosa Fast Flo^®^	1.4042	5.00	369
Comprecel^®^ 302	N.A.	N.A.	N.A.
Kollidon^®^ Va 64	N.A.	N.A.	N.A.
Microcel^®^ 102	N.A.	N.A.	N.A.
SeDeM			
Excipient	Weight (g)	Height (mm)	Hardness (N)
Emcompress^®^	0.9811	2.02	131
Lactosa Fast Flo^®^	1.0061	3.26	240
Comprecel^®^ 302	1.0206	3.63	451
Kollidon^®^ Va 64	0.7528	4.06	78
Microcel^®^ 102	0.8054	3.83	97

When the results are analysed, it is clear that low density excipients appear different from the others. First of all, it was not possible to obtain 5 and 4 mm tablets of Microcel^®^ 102, and neither was it possible for Kollidon^®^ Va 64 and Comprecel^®^ 302 at 5 mm. Moreover, Kollidon^®^ Va 64 and Microcel^®^ 102 increase their hardness when tablet height is reduced, which results are unexpected according to the literature, whereas Emcompress^®^ and Lactosa Fast Flo^®^ show decreased hardness when tablet thickness is reduced. Comprecel^®^ 302 could show the same trend although this is not appreciated in the graph. It was only possible to determine the hardness of Comprecel^®^ 302 at a height of 3.00 mm, 2.50 mm and 2.00 mm. Otherwise, the powder is so cohesive that the tablet’s hardness exceeds the device measurement limit (451 N).

The behaviour of Kollidon^®^ Va 64 and Microcel^®^ 102 is explained by the following reasoning. It is not possible to get a 1-gram tablet for both excipients even if the die is loaded at its maximum capacity. This means that the lower punch is at the lowest possible position when the compression force is applied. Thus, the minimum distance between the punches will depend on the upper punch’s maximum depth distance (12.00 mm in this study). When tablet weight is reduced, the lower punch rises. Then, if the upper punch penetrates the same distance in the die, the distance between the punches is reduced and the applied compression force rises. This implies that they were not being compressed at maximum possible force at some heights. According to the above, Kollidon^®^ Va 64 shows a decrease in hardness when tablet thickness is reduced to less than 3.00 mm where Kollidon^®^ Va 64 is already compressed at maximum compression force. Actually, we can conclude that it follows the same behaviour shown by the other excipients when it is compressed at its maximum compression force. However, Microcel^®^ 102 does not display a decrease in hardness because it is not yet compressed at maximum compression force at 2.50 mm, so its hardness is still increasing at 2.00 mm.

It should be pointed out that all the excipients, except Microcel^®^ 102, show a critical drop in hardness when thickness is reduced from 2.50 mm to 2.00 mm. On the other hand, the trend followed differs greatly between excipients when the powders are compressed at greater heights than 3.00 mm. Nonetheless, the range of heights between 2.50 mm and 3.00 mm shows a lower decrease in hardness (compared with the other ranges) and all the excipients (except Microcel^®^ 102) show a similar trend. As a result, it can be considered the best range to compare the cohesion of the excipients.

### Statistical analyses

The statistical study of the results supports the previous reasoning; an ANOVA Test was applied, see Table 5, as was a Fisher’s LSD, see Table 6.

**Table 5:** ANOVA outcome from the hardness results obtained.

Excipient	Source	Sum of squares	Degrees of freedom	Mean square	F coefficient	P value
Emcompress^®^	Between	104549.0	5	20909.8	137.4	<0.0001
	Within	12783.2	84	152.2	N.A.	N.A.
	Total	117332.2	89	N.A.	N.A.	N.A.
Lactosa Fast Flo^®^	Between	721215.0	5	144243.0	210.8	<0.0001
	Within	57468.3	84	684.2	N.A.	N.A.
	Total	778683.3	89	N.A.	N.A.	N.A.
Comprecel^®^ 302	Between	98793.4	2	49396.7	581.5	<0.0001
	Within	3567.7	42	84.9	N.A.	N.A.
	Total	102361.1	44	N.A.	N.A.	N.A.
Kollidon^®^ Va 64	Between	592523.0	4	148131.0	1758.8	<0.0001
	Within	5895.5	70	84.2	N.A.	N.A.
	Total	598418.5	74	N.A.	N.A.	N.A.
Microcel^®^ 102	Between	505431.0	3	168477.0	287.3	<0.0001
	Within	32838.0	56	586.4	N.A.	N.A.
	Total	538269.0	59	N.A.	N.A.	N.A.

**Table 6:** LSD test outcome of tablet hardness for each height.

	Differences					
Groups of contrast (mm)	Emcompress^®^	Lactosa Fast Flo^®^	Comprecel^®^ 302	Kollidon^®^ Va 64	Microcel^®^ 102	Sum of differences^2^
5.00–4.00	*37.2000	*-26.2000	N.A.	N.A.	N.A.	N.A.
4.00–3.00	*9.7333	*150.7330	N.A.^1^	*-202.1330	N.A.	N.A.
3.00–2.50	*16.8000	*20.2667	2.7300	*10.1333	*-117.4000	*167.3300
2.50–2.00	*31.8000	*92.0000	*98.0000	*77.4667	*-24.2000	*323.4667

^1^Comprecel 302’s tablet hardness was not determined due to hardness tester force limitation at heights of 4.00mm

^2^Sum of differences absolute values

*It indicates the existence of significant difference between these groups

ANOVA determines if there are significant differences between the studied tablet hardness for each height. A P-value below 0.0001 (obtained for all excipients) in the F-test means that there are significant differences between the means with a 95% confidence interval. As expected, these results demonstrate, statistically, that tablet dimensions affect tablet hardness. On the other hand, the Fisher’s LSD determines which means are significantly different with a 95% confidence interval and the estimated difference between the means. Even if there is not a common height range without significant differences, we can establish that the range between 2.50 mm and 3.00 mm is the best range to compare the cohesion of the excipients. The 3.00–5.00 mm range is discarded because it is not possible to compress two of five excipients in this range, due to their low bulk density. Consequently, the available ranges are 2.50–3.00 mm and 2.00–2.50 mm but if we analyse the sum of the differences (Table 6), the sum of differences in the range of 2.00–2.50 mm is almost twice the sum of differences in the range of 2.50–3.00 mm. Therefore, it can be concluded that tablet dimensions (height, in this case) affect tablet hardness in the range of 2.00–2.5 mm more than in the range of 2.50–3.00 mm.

With the relation established between density, weight and height (see Table 4), a new tablet weight adjustment is proposed in relation to powder density as long as the punch format is conserved: for excipients that have a bulk density lower than 0.40 g/cm^3^, the tablet weight is 0.720 g (± 20.0 mg) whereas for excipients with a bulk density greater than 0.70 g/cm^3^, the weight must be 1.225 g (±75.0 mg). One gram (± 50.0 mg) is maintained for excipients that show a bulk density between 0.40 g/cm^3^ and 0.70 g/cm^3^. To define the workable weight range at densities below 0.40 g/cm^3^, the average of Kollidon® Va 64 and Microcel® 102 tablet weight was calculated and rounded to only 2 decimal points at heights of 2.50 mm and 3.00 mm. The rounded weights are the lower limit and the upper limit whereas the average of both is the proposed weight. For the workable weight range at densities greater than 0.70 g/cm^3^, the same procedure was applied, though only the weight of Emcompress® was taken into account.

With this new weight adjustment, the Icd will be able to be determined for any kind of excipient, regardless of its bulk density, and the results will be accurate and comparable between them.

It should be noted that the Cohesion Index methodology is not limited to the punch format used in this study (19x10 mm oval convex). Whenever the punch format is changed, the weight of powder with bulk density from 0.40 to 0.70 g/cm^3^ should be calculated so as to keep the same mass per tablet volume. However, the weight increase (122.5%) and the weight reduction (72%) according the bulk density could be maintained.

### Application of SeDeM diagram: New methodology applied in formula design

As is well described in the literature, SeDeM diagram is a pharmaceutical method useful in a pharmaceutical development [1,10,11]. In this point, the impact of the improved Icd characterization in a formula development is shown.

**Fig 3:**
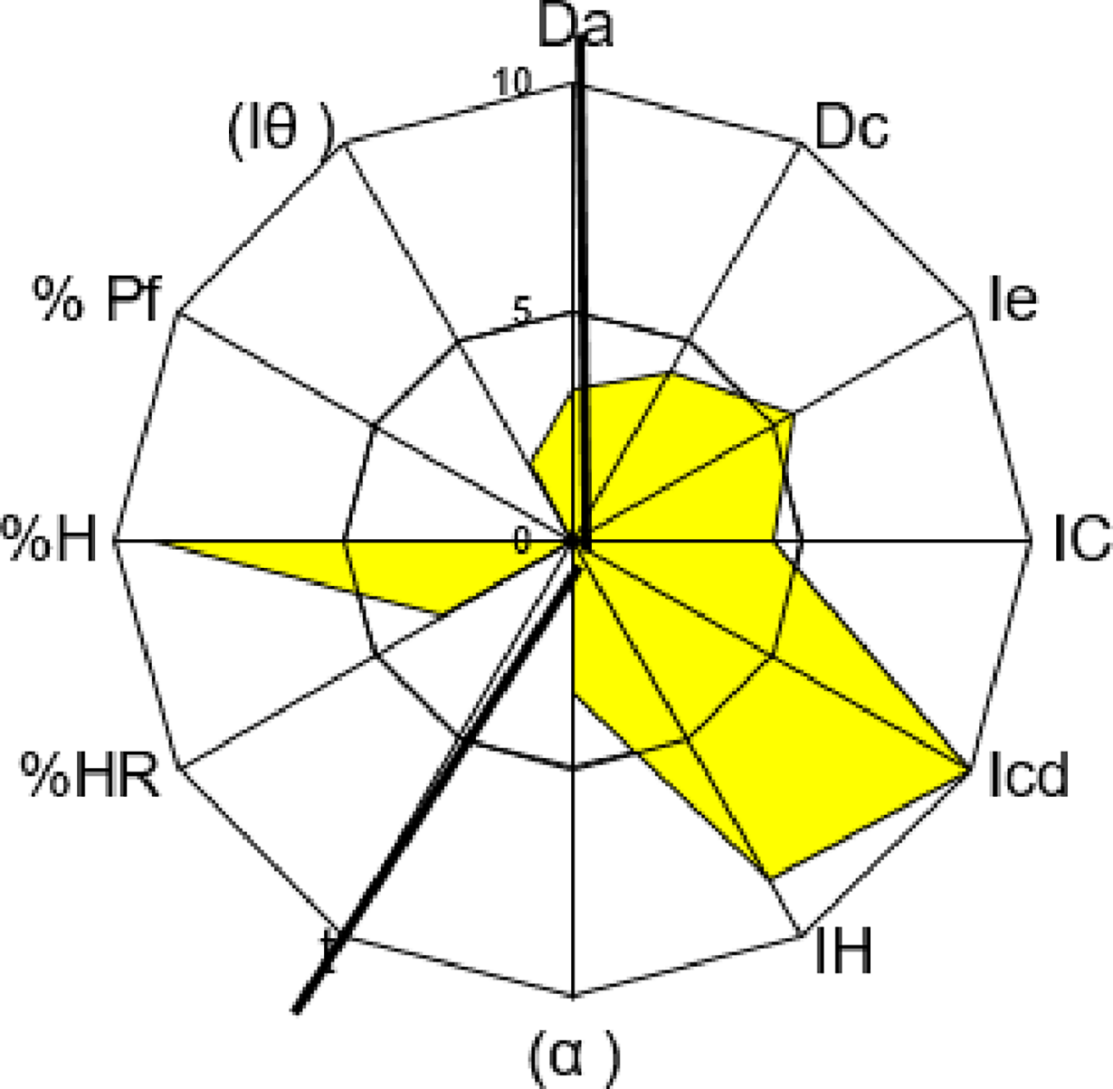
Microcel 102’s SeDeM diagram.

**Fig 4:**
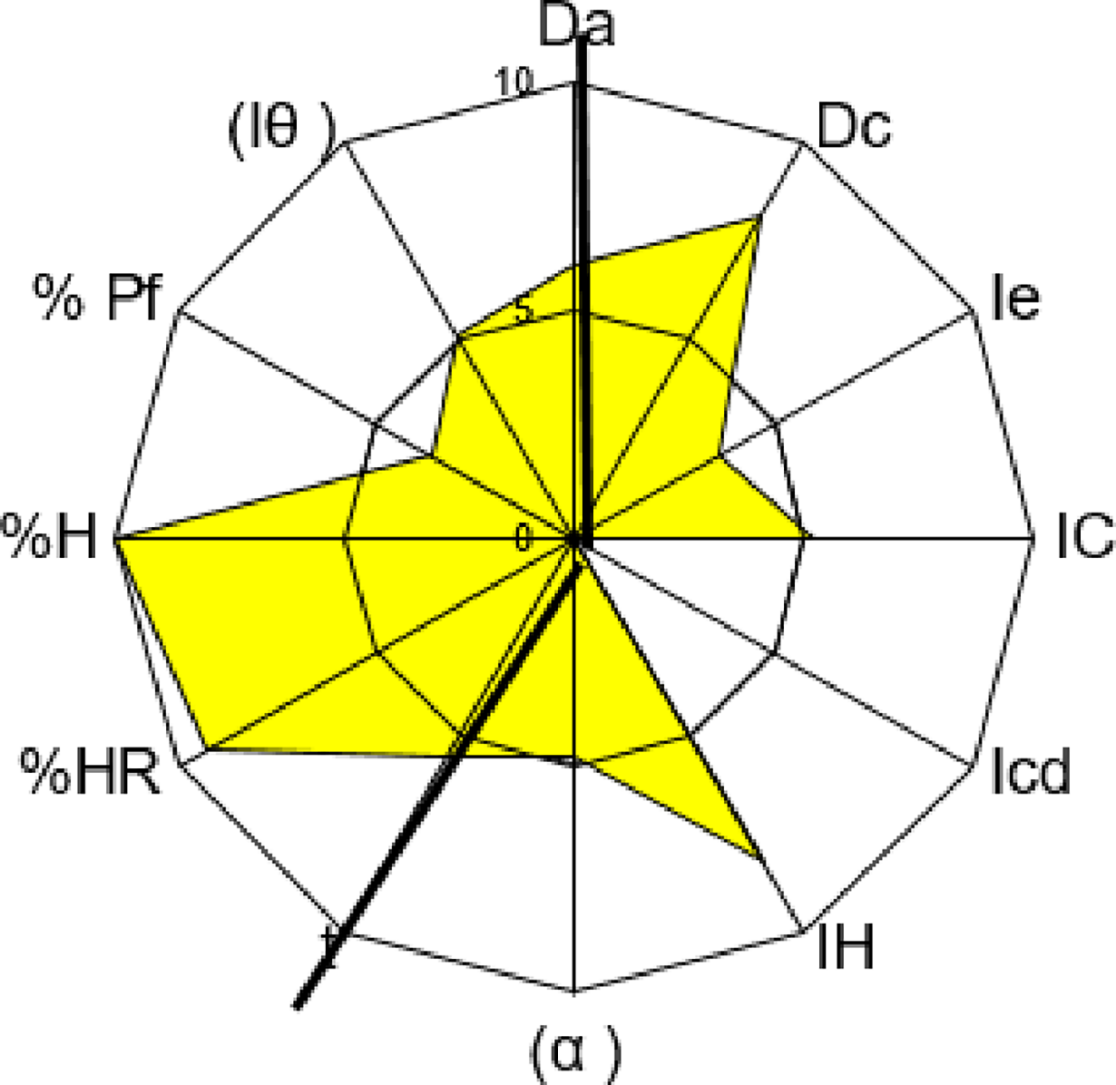
Sulfadimethoxine’s SeDeM diagram.

The drug substance selected for compression was Sulfadimethoxine, which has good properties for direct compression, although it does not have a good compressibility mean (see Table 3). Its properties make it a good tracer API to study compressibility. The excipient selected to correct Sulfadimethoxine’s deficiencies was Microcel 102, characterized and described previously, because MCCs are good as DC fillers. The SeDeM diagrams of Microcel 102 and Sulfadimethoxine are shown in Figs 3 and 4.

First of all, the amount needed to correct the active ingredient deficiencies is calculated with the following Eq 1 [6]:

$$CP=100-\left(\frac{RE-R}{RE-RP}\times 100\right),$$ (1)

Where CP is the % of corrective excipient, RE is the mean-incidence radius value (compressibility, in this case) of the corrective excipient, R is the mean-incidence radius value to be obtained in the blend, and RP is the mean-incidence radius value (compressibility, in this case) of the API to be corrected.

It should be pointed out that it would not be possible to correct the API’s deficiencies (low Icd) according to the previous SeDeM characterization [4,13], and 91.40% of Microcel 102 would be required to achieve a blend with a compressibility mean value of 4.75, as shown in Table 7. On the other hand, with the new proposed Icd determination only 55.80% of Microcel 102 is theoretically required to achieve an acceptable compressibility mean value (5.00).

**Table 7:** Theoretical amount of Microcel 102 to correct the API deficiencies.

		Previous SeDeM characterization		SeDeM characterization with Nm-Icd applied	
Incidence-mean	API	Microcel 102	Blend: expected radii values	Microcel 102	Blend: expected radii values
%	8.59	CP = 91.4	100	CP = 55.8	100
Dimensions	7.06	3.75	4.03	3.75	5.21
Compresibility	RP = 2.94	RE = 4.92	R = 4.75	RE = 6.63	R = 5.00
Flowability/Powder flow	6.20	3.98	4.17	3.98	4.94
Lubricity/Stability	9.65	6.28	6.57	6.28	7.77
Lubricity/Dosage	4.37	0.98	1.27	0.98	2.48
Average IPP	5.82	4.06	4.16	4.32	5.08

However, lubricants should be included in order to compress any powder automatically, so the following formula was designed: 53.88% of Microcel 102, 42.62% of Sulfadimethoxine, 0.14% of Colloidal Silicon R 200, 1.00% of Talc and 2.36% of Magnesium Stearate.

**Fig 5:**
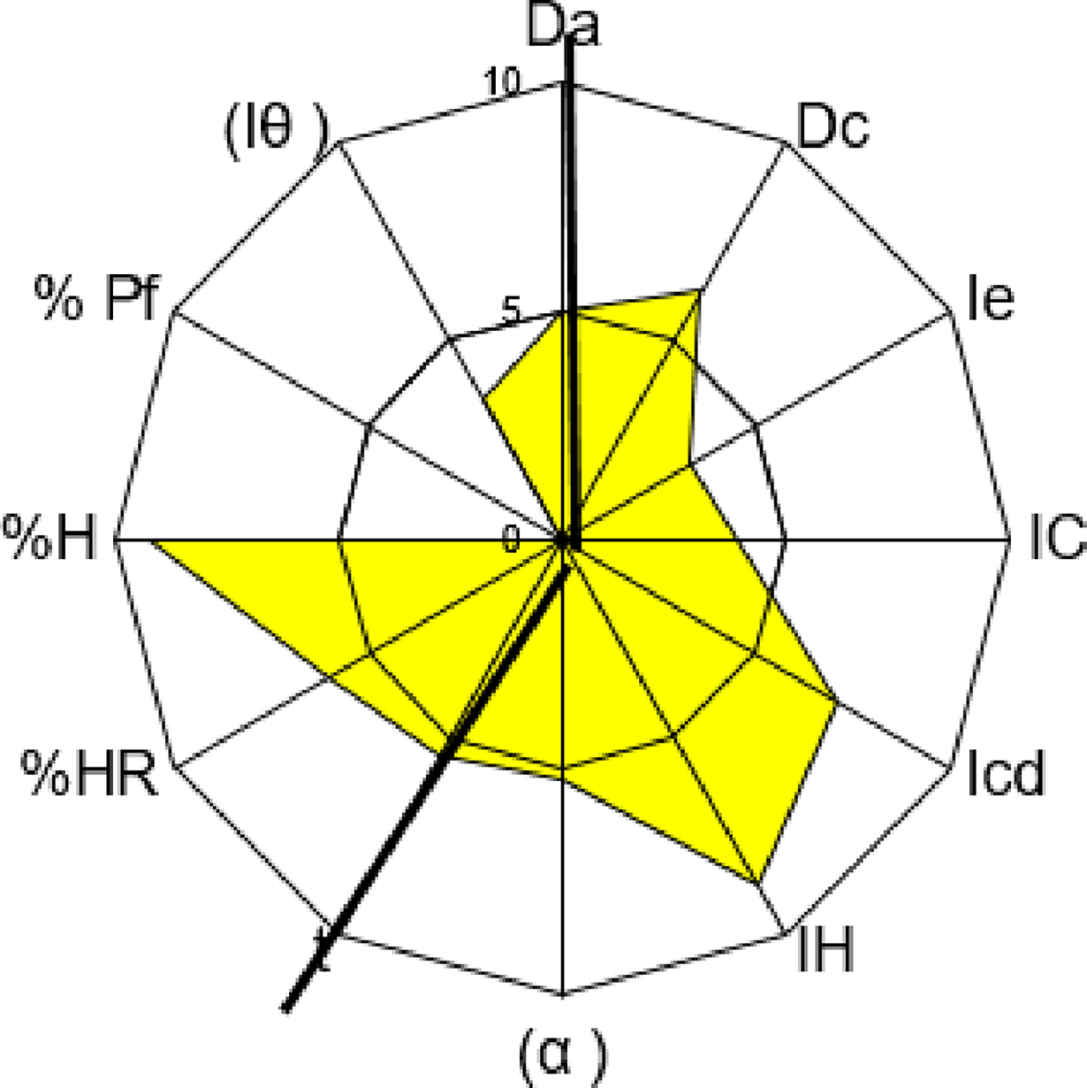
Final formula’s sedem diagram.

The blend was prepared and the parameter values were obtained following the described methodology. Each parameter was determined three times and the mean value was used for radius calculation. Diagram values obtained were then converted into radii (r) as described above. The corresponding parameters and the mean radius values obtained with the blend are shown in Table 8 and the diagram is shown in Fig 5.

**Table 8:** Final Formula parameters, incidence means and parametric index.

Incidence-mean	Parameter acronym	Experimental value	Radius value	Incidence mean value
Dimensions	Db	0.499 g/mL	4.99	5.61
	Dt	0.622 g/mL	6.22	
Compressibility	Ie	0.396	3.30	4.79
	IC	19.775%	3.96	
	Icd	142.4 N	7.12	
Flowability/Powder flow	IH	1.246	8.77	6.50
	(α)	23.642°	5.27	
	t”	9.087 s	5.46	
Lubricity/Stability	%HR	3.958%	6.04	7.62
	%H	1.592%	9.20	
Lubricity/Dosage	%Pf	58.282%	0.00	1.78
	(Iϴ)	0.0071	3.55	
Parametric Index	IP	0.58		
Parametric Profile Index	IPP	5.32		
Good Compression Index	IGC	5.07		

It is interesting that the determined incidence-means are quite close to the expected incidence-means, which means that both equation and newly proposed Icd determination methodology are accurate. The biggest difference is between expected and real flowability. This could be explained by the addition of lubricants considering them as gliding enhancers [21]. Recent studies also describe these little deviations as a consequence of the assumption of a linear behaviour by the SeDeM system [24].

Finally, the blend was compressed successfully and the described pharmacopoeia controls were performed according to their monographs. The results fulfil all the tests. For a mass uniformity the average mass is 1042.25 mg (max. mass: 1052.1 mg, min. mass: 1033.0 mg), they have an acceptable friability (0.52%), and for tablet hardness the average is 152 N (Max. hardness: 167, Min. hardness: 137). The pharmacopoeia controls demonstrate that it is possible to compress the proposed formula and obtain quality tablets.

## Conclusions

- It has been demonstrated that following the previous SeDeM methodology, excipients with a bulk density below 0.40 g/cm^3^ were not compressed at maximum compression force and excipients with a density higher than 0.72 g/cm^3^ were compressed at a height of 2.00 mm or less, which means that the results were not sufficiently accurate to be able to compare them.
- A new methodology to determine the Icd parameter is proposed: The powder is compressed at its highest compression force where it is possible to achieve a correct oval convex tablet of 19x10 mm. The weight of the tablet is adjusted concerning its bulk density: If the bulk density is higher than 0.70 g/cm^3^, the weight is 1.2250 (±0.075) and if it is smaller than 0.40 g/cm^3^, the weight is 0.7200 (±0.020). Otherwise, the weight is 1.0000 g (±0.050). Then, the hardness is determined for five tablets and, as a result, the radius value is calculated.
- With the improved characterization, it is possible to design a DC formulation of Sulfadimethoxine/Microcel 102/Lubricants using the equation, whereas it was not with the previous one. Therefore, the mathematical equation and the improved Icd characterization are accurate. Moreover, the final blend displayed similar incidence mean values to the expected values and the compression of that blend results in tablets which fulfil the pharmacopoeia tests.
